# Evaluation of two xenobiotic reductases from *Pseudomonas putida* for their suitability for magnetic nanoparticle‐directed enzyme prodrug therapy as a novel approach to cancer treatment

**DOI:** 10.1002/mbo3.1110

**Published:** 2020-09-26

**Authors:** Patrick Ball, Jennifer Halliwell, Simon Anderson, Vanessa Gwenin, Christopher Gwenin

**Affiliations:** ^1^ Bangor University Bangor UK; ^2^ Xi'an Jiaotong‐Liverpool University Suzhou China

## Abstract

Directed enzyme prodrug therapy (DEPT) is a cancer chemotherapy strategy in which bacterial enzymes are delivered to a cancer site before prodrug administration, resulting in prodrug activation at the cancer site and more localized treatment. A major limitation to DEPT is the poor effectiveness of the most studied enzyme for the CB1954 prodrug, NfnB from *Escherichia coli*, at concentrations suitable for human use. Much research into finding alternative enzymes to NfnB has resulted in the identification of the Xenobiotic reductases, XenA and XenB, which have been shown in the literature to reduce environmentally polluting nitro‐compounds. In this study, they were assessed for their potential use in cancer prodrug therapy strategies. Both proteins were cloned into the pET28a+ expression vector to give the genetically modified proteins XenA‐his and XenB‐his, of which only XenB‐his was active when tested with CB1954. XenB‐his was further modified to include a cysteine‐tag to facilitate direct immobilization on to a gold surface for future magnetic nanoparticle DEPT (MNDEPT) treatments and was named XenB‐cys. When tested using high‐performance liquid chromatography (HPLC), XenB‐his and XenB‐cys both demonstrated a preference for reducing CB1954 at the 4‐nitro position. Furthermore, XenB‐his and XenB‐cys successfully induced cell death in SK‐OV‐3 cells when combined with CB1954. This led to XenB‐cys being identified as a promising candidate for use in future MNDEPT treatments.

## Graphical abstract

Both recombinant XebB‐his and XenB‐cys successfully induced cell death in SK‐OV‐3 cells when combined with the nitroaromatic prodrug CB1954. This led to XenB‐cys being identified as a promising candidate for use in cancer prodrug therapy treatments.

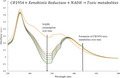

## 1 INTRODUCTION

Traditional cancer treatment methods, including surgery, radiotherapy, and chemotherapy, have progressed over recent years; however, there are still many limitations associated with these treatments such as the lack of selectivity between healthy and cancerous cells (Braun & Seymour, 2011; Brown & Giaccia, 1998). One approach being investigated to improve the selectivity of chemotherapy treatments is to direct the treatment to the cancer site, as is the case in directed enzyme prodrug therapy (DEPT) (Kratz, Müller, Ryppa, & Warnecke, 2008; Sinhababu & Thakker, 1996), which involves the delivery of a prodrug‐activating enzyme to the cancer site before prodrug administration. Methods of directing prodrug‐activating enzymes to solid tumors include antibodies (ADEPT) (Bagshawe, 2006), genes (GDEPT) (Denny, 2001; Williams et al., 2015), viruses (VDEPT) (Chung‐Faye et al., 2001; McNeish et al., 1998; Palmer et al., 2004; Race et al., 2007), and gold‐coated magnetic nanoparticles (MNDEPT) (Gwenin, Gwenin, & Kalaji, 2009, 2011).

Bacterial nitroreductases (NTRs) are one type of prodrug‐activating enzyme used in DEPT strategies due to their ability to reduce the nitroaromatic prodrug CB1954 to its cytotoxic derivatives (Knox, Boland, et al. (1988); Knox, Friedlos, Jarman, & Roberts, 1988; McNeish et al., 1998; ; ; Knox, Friedlos, Marchbank, & Roberts, 1991; Weedon et al., 2000; Searle et al., 2004; Vass et al., 2009; Patel et al., 2009). Bacterial NTRs reduce prodrugs to their active forms following a two‐electron transfer in the presence of a NAD(P)H cofactor (Bryant & DeLuca, 1991; Bryant, Hubbard, & McElroy, 1991; Bryant, McCalla, Leeksma, & Laneuville, 1981; De Oliveira, Bonatto, Antonio, & Henriques, 2010; Prosser et al., 2013). This reaction follows a ping‐pong bi‐bi reaction mechanism where the NAD(P)H cofactor donates electrons to the FMN prosthetic group, reducing it, allowing the enzyme to reduce the prodrug to its active form (Chou & Talalay, 1977; Gwenin et al., 2015; Valiauga, Williams, Ackerley, & Cenas, 2016; Zenno, Koike, Tanokura, & Saigo, 1996). The most heavily investigated NTR for use in DEPT strategies is the NfnB NTR from *Escherichia coli* (*E*. *coli*) which has been shown to reduce CB1954 to either the 2‐ or 4‐hydroxlyamine (NHOH) metabolites (Gwenin et al., 2015; Prosser et al., 2013; Williams et al., 2015), with the 4‐NHOH product being shown to form DNA crosslinking species intracellularly upon reacting with cellular thioesters (Emptage, Knox, Danson, & Hough, 2009; Gwenin et al., 2015; Prosser et al., 2013; Race et al., 2007). The low turnover rate of CB1954 by NfnB has proven to be a significant limitation for this combination, so finding new ways to improve this clinical approach is of the utmost importance (Chung‐Faye et al., 2001; Gwenin et al., 2015; Searle et al., 2004).

Approaches to overcoming the limitations of the NfnB/CB1954 combination have included the development of other prodrugs with greater dose potency such as the hypoxia‐activated prodrugs PR‐104A (Foehrenbacher et al., 2013; Guise et al., 2010; McKeage et al., 2011; Patterson et al., 2007; Singleton et al., 2009; Williams et al., 2015) and SN27686 (Singleton et al., 2007) or the identification of other bacterial enzymes that operate in combination with the CB1954 prodrug such as NfsA from *E*. *coli* (Copp et al., 2017; Vass et al., 2009), YfkO from *Bacillus licheniformis* (Emptage et al., 2009), or the novel nitroreductases from *Bacillus cereus*, BC_1619 and BC_3024, previously identified within our research group (Gwenin et al., 2015).

The two Xenobiotic reductases, XenA and XenB, from *Pseudomonas putida* (*P*. *putida*) have been identified from the literature as being capable of reducing a nitro (NO_2_) group, such as those found on explosive compounds like nitro‐glycerin, in the presence of an NADPH cofactor to produce an NHOH derivative (Blehert, Fox, & Chambliss, 1999; Orville, Manning, Blehert, Fox, & Chambliss, 2004; Orville, Manning, Blehert, Studts, et al., 2004; Pak et al., 2000). Like NfnB, the two Xenobiotic reductases are flavoproteins but, unlike NfnB, which is a dimeric flavoprotein, they are monomeric flavoproteins (Orville, Manning, Blehert, Fox, et al., 2004; Orville, Manning, Blehert, Studts, et al., 2004). While the ability of the XenA and XenB enzymes to reduce to nitro groups in explosive compounds in the presence of NADPH is well documented within the literature, their activity with nitroaromatic prodrugs such as CB1954 is yet to be established.

In this study, it was determined whether or not the two Xenobiotic reductases were able to readily reduce the CB1954 prodrug to its toxic metabolites to ascertain their potential for use in novel MNDEPT treatments. Firstly, for the ease of purification and isolation, both Xenobiotic reductases were modified to contain a His‐tag, and only the enzyme which showed reactivity toward the CB1954 prodrug, XenB, was then further modified to contain an N‐terminal Cys‐tag for immobilization onto gold‐coated magnetic nanoparticles (Gwenin, Gwenin, & Kalaji, 2011) for possible use in MNDEPT.

## 2 MATERIALS AND METHODS

### 2.1 Cloning of enzymes

Genomic DNA was isolated from *P. putida* using a Wizard® Genomic DNA Purification kit from Promega. The primers listed in Table 1 were synthesized by Eurofins Genomics and used to amplify the *xenA* and *xenB* genes from the genomic DNA using a Techne TC‐3000 thermocycler. Amplification of the *xenA* and *xenB* genes from genomic DNA was achieved using PCR set up using thermic cycling parameters under the following conditions; 98°C for 30 s, 15 cycles of 98°C for 10 s (denaturing), 68–58°C for 30 s (annealing), and 72°C for 30 s (extension). This was then followed by a final hold at 4°C. The PCR products were purified as previously described (Gwenin et al., 2015) using a GeneJet PCR purification kit and subjected to a restriction enzyme digest using the sites indicated in Table 1 before being analyzed using a 1% Agarose gel to confirm their size and purity.

**TABLE 1:** Primer sequences used for the cloning of XenB and XenA

Primer name	Sequence (5′ → 3′)
*xenA* Fw	GAGTTT*CATATG*TCCGCACTGTTC
*xenA* Rv	GCAG*GTCGA*CCAAGCCTCAGC
*xenB* Fw	TAACC*CATATG*ACCACGCTTTTCGATCC
*xenB*‐cys Fw	AGGTA*GGATCC* **TGTTGTTGTTGCTGTTGC**ATGACCACGCTTTT
*xenB* Rv	GAAT*GTCGAC*CAATCACAACCGCGGATA

Note

The restriction enzyme cut site is in italics and underlined. The addition of the cysteine‐tag is in bold and underlined.

After being purified, ligation between the PCR products and a pET28a^+^ vector (Novagen, Merck, UK) was performed using t4 DNA ligase (New England Biolabs, UK) at 16°C overnight. To confirm successful ligation between the gene of interest and the plasmid vector, a PCR based on the T7 promoter and T7 terminator sequences, which flank the gene insert region contained within the vector, was performed using Taq DNA Polymerase Master Mix (Amplicon, Denmark). To amplify the recombinant plasmids, competent *E*.* coli* DH5α cells (200 µl) were transformed with a plasmid (~10 µl) and incubated on agar plates containing Kanamycin (50 µg/ml). Kanamycin is used to select for bacterial colonies containing the pET28a^+^ plasmid with the Kan^R^ gene.

### 2.2 Transformation

The transformation of recombinant plasmids containing the cloned *xenA* and *xenB* genes into *E*. *coli* Rosetta pLysS (Novagen, Merck, UK) was done following the method described previously by Ball, Thompson, Anderson, Gwenin, and Gwenin (2019).

### 2.3 Protein expression and purification

The expression and purification of all proteins used were done following the method previously described by Ball et al. (2019), with one minor modification. In this work, all Lysogeny Broth media used contained additional glucose (0.5%).

### 2.4 Enzyme reactivity with CB1954

The ability of the purified enzymes to reduce the CB1954 prodrug was confirmed following the method previously described by Gwenin et al. (2011). Briefly, the proteins were incubated with NADPH (300 μM) and CB1954 (100 μM) in phosphate buffer (PB) (50 mM, pH 7.2) and scanned using UV‐visible spectroscopy every 90 s for 15 min. For active enzyme/CB1954 combinations, product formation was measured at 420 nm (Gwenin et al., 2011, 2015).

### 2.5 CB1954 kinetics

All enzyme kinetics experiments were carried out following the method previously described by Ball et al. (2019), using a Thermo Scientific Varioscan 96‐well plate microplate reader. The data were then transferred to SigmaPlot 12 (SPSS, Systat Software Inc.) where a non‐linear regression tool was used to generate a Michaelis–Menten hyperbolic curve and a report containing the important kinetic information of the system under test.

### 2.6 HPLC

All of the HPLC experiments in this study were carried out following the method previously described by Ball et al. (2019), using an HPLC machine (Dionex Ultimate 3000 HPLC system, ThermoScientific, USA) using a C18 column for analysis (Waters Spherisorb® 5 µm ODS2 4.6 mm × 250 mm C18 column, UK). The instrument was run using the following parameters: 50 µl injection volume, a fixed column oven temperate of 25°C, a run length of 45 min, and the UV wavelength for detection was 420 nm (Ball et al., 2019; Gwenin et al., 2015).

### 2.7 Cell viability assays

Cell viability experiments were conducted using an MTT assay (Mosmann, 1983), following the method previously described by Gwenin et al. (2015). Briefly, SK#x2010;OV‐3 cells (Sigma–Aldrich, United Kingdom) were seeded at a density of 1000 cells per well, in 100 µl Dulbecco's Modified Eagles Medium (DMEM) containing 10% FBS and 1% penicillin/streptomycin and were allowed to attach overnight in a 5% CO_2_ incubator at 37°C. After 16 h, the medium was aspirated, and fresh medium (50 µl) containing CB1954 (20 µM) was added. Next, medium (50 µl) containing the purified enzyme was added and after 4 h, the medium was removed, and cells were replenished with complete DMEM (100 µl). After 48 h, 20 µl of MTT (5 mg/ml) was added to each well and incubated at 37°C for 4 h. The formazan crystals that formed were dissolved in 100 µl of DMSO, after removing the media carefully, and the absorbance was read at 570 nm in a Thermo Scientific Varioscan 96‐well plate microplate reader.

## 3 RESULTS

### 3.1 Cloning and sequencing

Both the *xenA* and *xenB* genes were successfully amplified using PCR, purified, and inserted into the pET28a^+^ expression vector, which adds an N‐terminal histidine tag (his‐tag) for ease of protein purification. The recombinant plasmids containing the *xenA* and *xenB* genes were sequenced verified to confirm their identity before they were used in other experiments.

### 3.2 Protein expression and purification

The pET28a+ vector contains a his‐tag that is added to all the recombinant proteins for ease of purification using metal ion affinity chromatography (IMAC). XenA‐his and XenB‐his are the native *P. putida* XenA and XenB cloned into the pET28a+ vector. The XenB‐cys, however, was genetically modified using PCR to contain an additional cysteine‐tag (designed by our research group) (Gwenin et al., 2011) on the N‐terminal of the protein which is 6 amino acids long. The purpose of the cys‐tag is to allow direct binding of the protein to metal surfaces such as gold. The additional weight of the amino acid tags causes small changes in migration on the SDS‐PAGE as previously described (Gwenin et al., 2011). All genetically modified proteins were obtained at a yield of up to 5 mg/ml.

While NTR genes express a homodimeric flavoprotein (Gwenin et al., 2015; Parkinson, Skelly, & Neidle, 2000) (NfnB monomer unit approximately 26 kDa (Gwenin et al., 2015)), the Xenobiotic reductase genes express a monomeric flavoprotein (Orville, Manning, Blehert, Fox, et al., 2004; Orville, Manning, Blehert, Studts, et al., 2004) with XenA having a unit size of roughly 39 (Orville, Manning, Blehert, Studts, et al., 2004) kDa and XenB having a unit size of approximately 40 kDa (Orville, Manning, Blehert, Fox, et al., 2004).

### 3.3 Enzymatic reduction of the CB1954 prodrug

**FIGURE 1:**
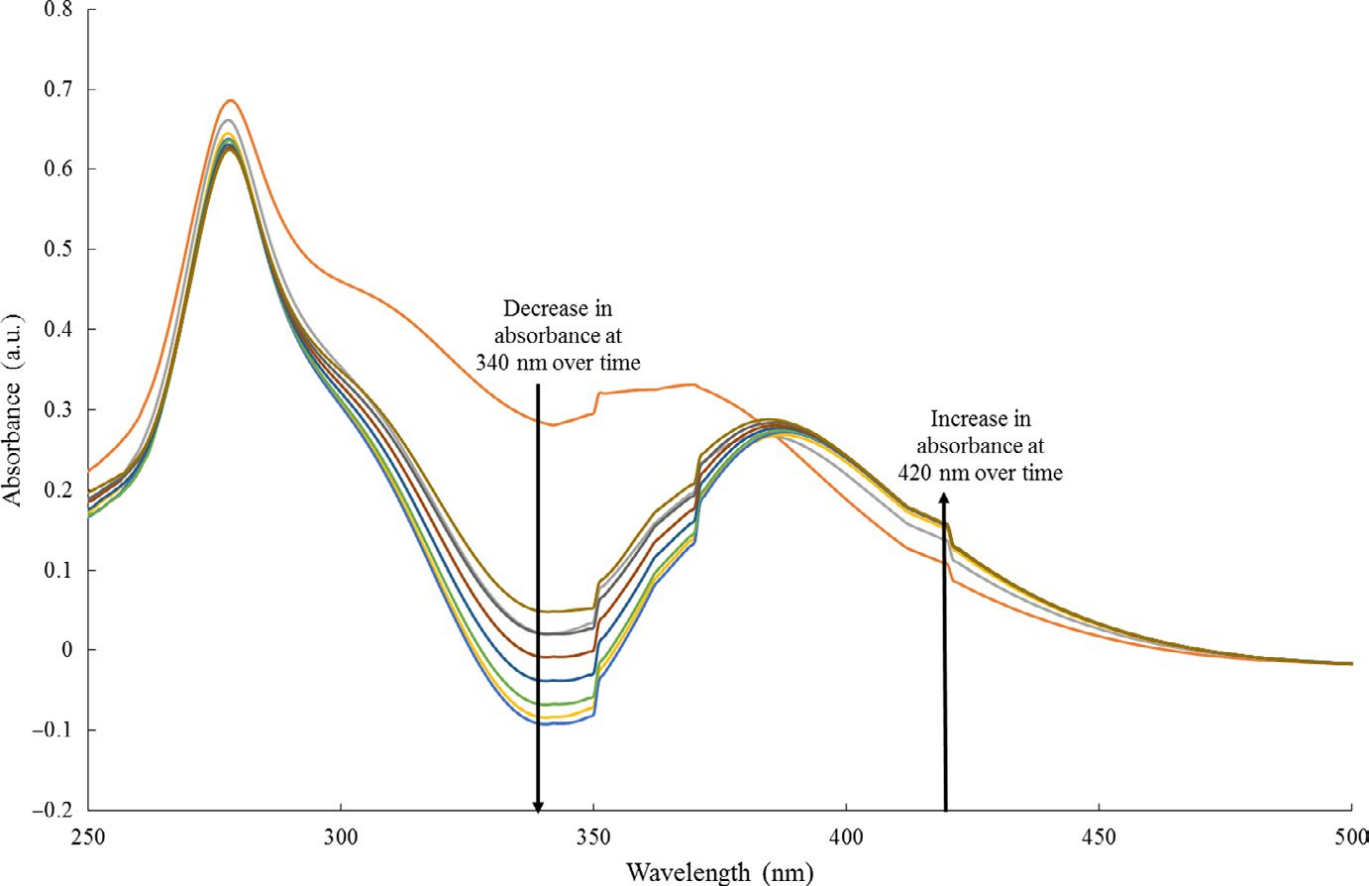
UV/Vis spectra showing the enzymatic reduction of CB1954 to its hydroxylamine derivatives (λ_max_ = 420 nm) by XenB‐his in presence of NADPH (λ_max_ = 340 nm)

Initially, the reactivity of each enzyme with CB1954 in the presence of an NADPH cofactor was tested (Figure 1) following the method as previously described by V. Gwenin et al. (2011), Gwenin et al. (2015). For active enzyme/prodrug combinations, NADPH consumption was observed at 340 nm and the formation of the CB1954 hydroxylamine products was seen at 420 nm.

XenB‐his and XenB‐cys both demonstrated the ability to reduce the CB1954 using an NADPH cofactor; however, XenA‐his showed no reactivity with CB1954. To test the NO_2_‐reducing ability of XenA‐his against work found in the literature (Yanto, Yu, Hall, & Bommarius, 2010), it was tested again with a nitro‐glycerin substrate and, in this instance, it was able to reduce the NO_2_ groups present in the substrate. It was postulated that the XenA‐his enzyme is unable to reduce a nitroaromatic substrate such as CB1954, as literature has also shown an inability of the XenA enzyme to reduce the nitroaromatic explosive trinitrotoluene (TNT) in a similar manner (Pak et al., 2000), and it was decided not to test the XenA‐his enzyme any further at this stage, but to only proceed with the recombinant XenB‐his and XenB‐cys.

Thus, the Michaelis–Menten kinetic parameters were only determined for XenB‐his and XenB‐cys with respect to varying concentrations of CB1954 (Table 2).

**TABLE 2:** The Michaelis–Menten kinetic data obtained for XenB‐his and XenB‐cys (10 µg/ml) by varying the concentrations of the CB1954 prodrug in the presence of NADPH as the cofactor

Enzyme	Apparent Vmax μM S^−1^	Apparent Km μM	Apparent Kcat S^−1^	Apparent Kcat/Km μM^−1^ s^−1^
XenB‐his	5.9	1690	8.2	0.0049
XenB‐cys	2.5	457	1.8	0.0039
NfnB‐his	22.9	4064	25.1	0.0062

Note

Previously published data for NfnB‐his have also been shown for comparison to XenB‐his and XenB‐cys (Ball et al., 2019).

When comparing the apparent Michaelis–Menten kinetic parameters obtained for XenB‐his and XenB‐cys reacting with CB1954, it appeared that the incorporation of the cysteine‐tag has led to a decrease in the kinetic activity of the enzyme. XenB‐cys demonstrated a lower turnover of CB1954 (Kcat = 1.8 s^−1^ compared to 8.2 s^−1^ for XenB‐his) and a lower efficiency in its reaction with the prodrug compared to XenB‐his (Kcat/Km = 0.0039 μM^−1^ S^−1^ compared to 0.0049 μM^−1^ S^−1^ for XenB‐his) despite having a much higher affinity for the prodrug (Km = 457 μM compared to 1690 μM for XenB‐his). When compared to the kinetic data observed when testing NfnB‐his with the CB1954 prodrug, it is evident that both XenB‐his and XenB‐cys demonstrate a superior affinity for the prodrug as they display a much lower value for Km (Km = 4064 μM for NfnB‐his). However, both XenB‐his and XenB‐cys were shown to be less efficient in their reaction with CB1954 than NfnB‐his as they were shown to have lower values of Kcat/Km (Kcat/Km = 0.0062 μM^−1^ s^−1^ for NfnB‐his). The lower Km values produced when using XenB‐his and XenB‐cys compared to NfnB‐his is a result of clinical significance as the maximum tolerated dose of CB1954 in humans is less than 10 μM CB1954 (Chung‐Faye et al., 2001); therefore, enzymes that operate better at lower prodrug concentrations are likely to be viable in a clinical setting.

### 3.4 HPLC analysis

**FIGURE 2:**
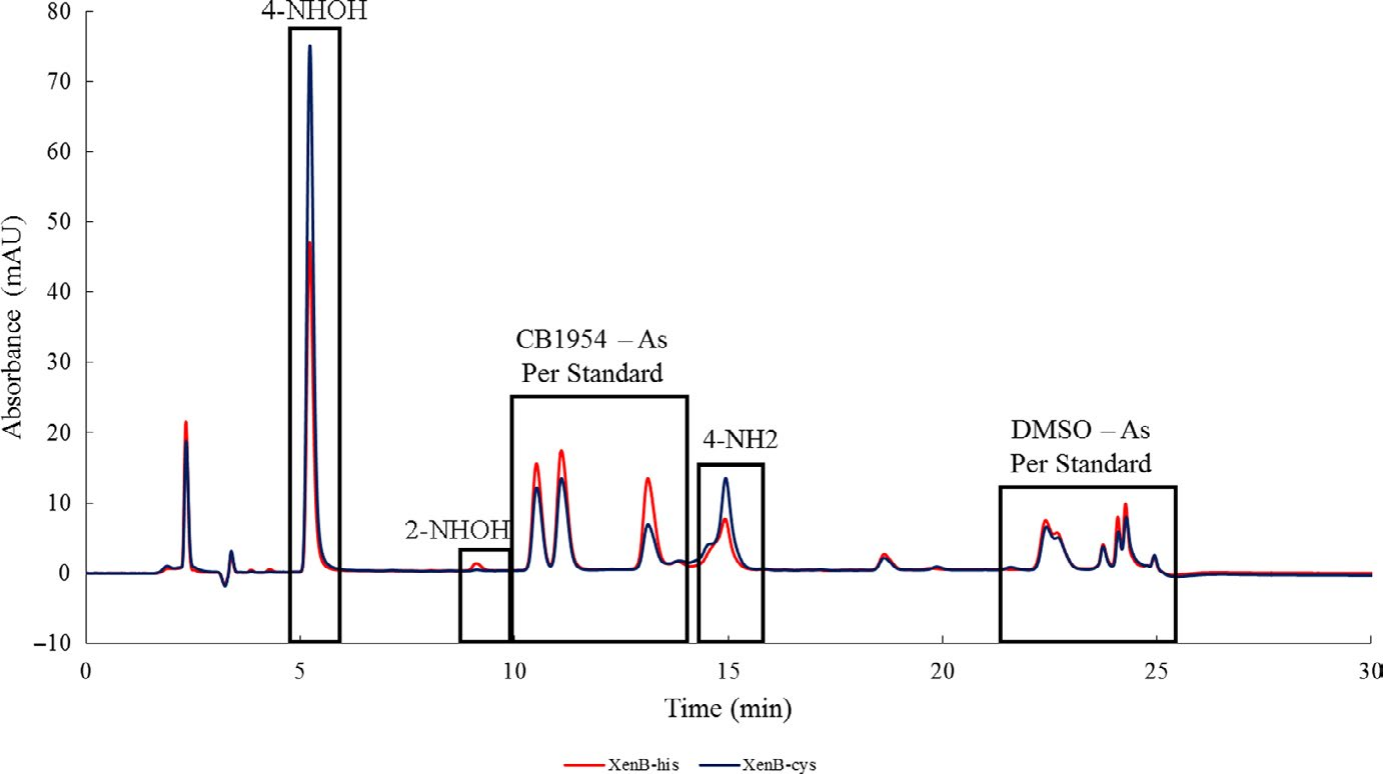
HPLC chromatogram of the reaction between CB1954 and either XenB‐his or XenB‐cys, measuring absorbance at 420 nm. NADPH and phosphate buffer were detected at 2–4 min, unreacted CB1954 prodrug was detected at 10.5–14 min and DMSO was detected at 22.5–24.5 min. The 4‐hydroxylamine was detected at 5–6 min and the corresponding amine was detected at 14.5–15.5 min. The 2‐hydroxylamine was detected at 9–10 min with the corresponding amine being detected at 21–22 min

Following the method previously published (Ball et al., 2019; Gwenin et al., 2015), the ratio of the CB1954 NHOH derivatives formed after the reduction of the CB1954 prodrug by either XenB‐his or XenB‐cys (Figure 2) was analyzed and these ratios are presented in Table 3.

**TABLE 3:** The ratio of the CB1954 hydroxylamine derivatives formed when the prodrug is reacted with different enzymes

Enzyme	Hydroxylamine product ratio (2‐NHOH:4‐NHOH)
XenB‐his	3:97
XenB‐cys	6:94

The hydroxylamine product ratios produced for XenB‐his (3:97) and XenB‐cys (6:94) were both extremely similar to one another, and in both cases, the enzyme demonstrated a preference for reducing CB1954 at the 4‐position to give the 4‐NHOH as the major product of the reaction. This is a result of clinical significance as the 4‐NHOH derivative of CB1954 has been shown in the literature to be further reduced by intracellular thioesters such as Acetyl Coenzyme A to form a compound that can cross‐link DNA (Anlezark et al., 1992; Knox et al., 1991; Race et al., 2007).

### 3.5 Cell viability assays

**FIGURE 3:**
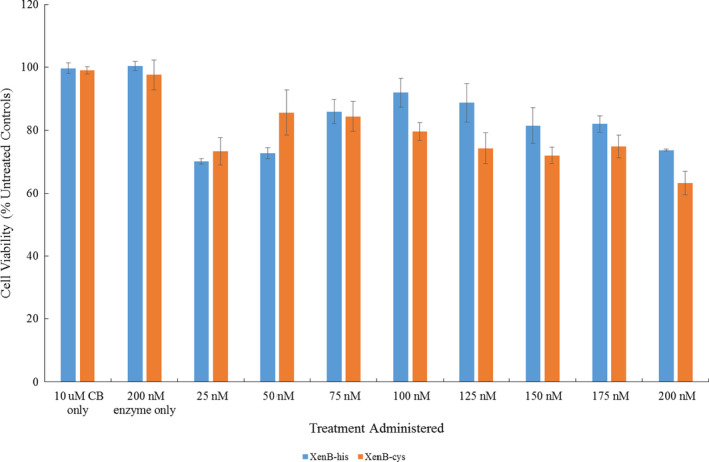
Percentage cell survival relative to untreated control cells of SK#x2010;OV‐3 cells after a 4 h incubation with prodrug only, the enzyme only and increasing concentrations of either XenB‐his or XenB‐cys (25–200 nM) in presence of CB1954 (10 µM). All data points are taken from the averages of at least three repeats, and the error bars represent the standard deviation

Percentage cell viability of SK#x2010;OV‐3 cells, relative to untreated controls, was determined in the presence of increasing concentrations of either XenB‐his or XenB‐cys enzyme, with a constant concentration of CB1954 present (Figure 3). Controls were cell culture medium only, the enzyme only and prodrug only and data points were plotted based on the averages taken from at least three repeats. The CB1954 concentration used was fixed at 10 µM so that the results of the cell viability assays could be related to clinical results, because the CB1954 concentration in vivo cannot exceed 10 µM due to the maximum tolerated dose observed in clinical trials thus far (Chung‐Faye et al., 2001; McNeish et al., 1998; Searle et al., 1998, 2004; Williams et al., 2015).

Promisingly, no cell kill was observed when cells were treated with the controls of CB1954 or enzyme. Upon the addition of 25 nM enzyme, both for XenB‐his and XenB‐cys, there was an immediate response in terms of cell viability percentage with an observed cell kill of roughly 25%–30%. As there was no added NADPH cofactor present in the cell viability assay, it is of promise that such a significant cell kill indicated that the enzymes, free in solution, were taken up into the SK#x2010;OV‐3 cells. It was noticeable that there occurred an initial decrease in the cell‐killing potency of treatments as the enzyme concentration was increased from 25 nM before the cell‐killing potency began to increase again as the enzyme concentration approached 200 nM. While this may initially appear to be an unexpected result, it is a known phenomenon called the Hormetic effect, which has been well documented in the literature (Mattson, 2008). The Hormetic effect is evident in the biphasic response of the SK#x2010;OV‐3 cells to the increasing amounts of enzyme used in the treatments, leading to the characteristic inverted U‐shaped response on the graph (Mattson, 2008). Also seen from Figure 3 was that neither enzyme nor the CB1954 prodrug alone could cause significant cell death.

## 4 DISCUSSION

The major aim of this research was to clone and express genetically modified Xenobiotic reductases from *P. putida* and assess their ability to reduce the CB1954 prodrug and demonstrate an ability to cause cell death in the ovarian cancer cell line, SK‐OV‐3. It is of significance that these enzymes have never before been reacted with the Cb1954 prodrug, and it was hoped that the results obtained would show the recombinant enzymes to be suitable candidates for use in the novel‐directed enzyme prodrug therapy approach, MNDEPT (Gwenin, Gwenin, & Kalaji, 2009; Gwenin et al., 2011). To achieve the aims, it was determined whether or not the enzymes could (a) effectively reduce CB1954 at low prodrug concentrations, (b) had a preference for reducing the prodrug at the 4‐NO_2_ position instead of the 2‐NO_2_ position, and (c) cause cell death in Sk‐OV‐3 ovarian cancer cells when reacted with the prodrug.

All of the aims were achieved and XenB‐cys identified as a promising candidate for MNDEPT. Despite the poor activity of XenA‐his leading to this enzyme being dropped from the study, XenB‐his and subsequently XenB‐cys have been shown to reduce the CB1954 prodrug. Both proteins showed a preference for reduction at the more desirable 4‐NO_2_ position and induced increased cell death in the ovarian cancer cell line SK‐OV‐3.

The fact that XenB‐his and XenB‐cys were shown to reduce CB1954 almost exclusively at the 4‐NO_2_ position was a significant result because intracellularly the 4‐NHOH derivative of CB1954 is further reduced by thioesters to a DNA crosslinking species which is highly cytotoxic (Anlezark et al., 1992). It has been shown previously by Ball et al. (2019) that the hydroxylamine product ratio produced after the reduction of the CB1954 prodrug changes over time in favor of the 4‐NHOH product as the 2‐NHOH product is more rapidly converted *via* a non‐enzymatic reduction into the corresponding amine (2‐NH_2_) than the 4‐NHOH product. In the case of XenB‐his and XenB‐cys, a negligible amount of 2‐NH_2_ was obtained, which indicated that little‐to‐no 2‐hydroxylamine had been formed and that both proteins reduced CB1954 almost exclusively at the 4‐NHOH position. It should be noted that while reduction at the 4‐NO_2_ position is more favorable in terms of generating a more potent DNA‐crosslinking product, literature has suggested that there may also be negatives to using enzymes that favor 4‐NO_2_ reduction over 2‐NO_2_ reduction because of the significantly higher bystander effect of the products formed after CB1954 is reduced at the 2‐NO_2_ position (Chan‐Hyams, Copp, Smaill, Patterson, & Ackerley, 2018; Helsby, Ferry, Patterson, Pullen, & Wilson, 2004; Vass et al., 2009).

Upon analysis of the kinetic data obtained for XenB‐his and XenB‐cys, both proteins yielded a lower value for Km than has been previously reported in the literature for the heavily investigated NfnB NTR. Again this result is of clinical significance as any enzyme used in cancer prodrug therapy treatments in combination with CB1954 must be effective at lower prodrug combinations due to the dose‐limiting toxicity of CB1954 restricting the concentration of the prodrug which can be used in treatments.

As well as characterizing the reaction between the Xenobiotic reductases, XenB‐his or XenB‐cys, with the CB1954 prodrug, it was important to ascertain if the enzyme/prodrug combinations could cause cell death in a cancerous cell line, in this case, the ovarian cancer cell line SK#x2010;OV‐3. It was decided that the cancer cell kill experiments would be carried out without the addition of extracellular cofactor so that any cell‐killing observed could be attributed to the successful uptake of the enzymes into the cancer cells, thus accessing the intracellular cofactor. The results obtained for the 2D cell culture experiments confirmed that XenB‐his and XenB‐cys were successfully taken up into SK#x2010;OV‐3 cells and induced cell death after reducing the CB1954 prodrug to its toxic products, thus identifying them as promising enzymes for use in cancer prodrug therapy treatments.

In conclusion, two Xenobiotic reductases were identified, cloned, expressed, and tested with the CB1954 prodrug. Unfortunately, the XenA‐his reductase was shown not to reduce the CB1954 prodrug at detectable levels, even though it was capable of reducing nitro‐glycerin. Together with other literature, it was thus hypothesized that this enzyme was unable to reduce nitroaromatic compounds. XenB‐his and subsequently XenB‐cys were shown to be able to readily reduce CB1954, primarily at the 4‐NO_2_ position, and these enzyme/prodrug combinations were shown to cause significant cell death in SK‐OV‐3 cells without the addition of extracellular cofactor thus confirming they are promising candidates for use in DEPT strategies. The next stage of this research will be to assess the XenB‐cys enzyme when immobilized onto gold‐coated magnetic nanoparticles (AuMNPs) to see if this leads to any loss of activity with the CB1954 prodrug or a change in the performance in cell viability assays.

## CONFLICT OF INTEREST

None declared.

## AUTHOR CONTRIBUTIONS

**Patrick Ball:** Formal analysis (equal); writing – original draft (lead). **Jennifer Halliwell:** Formal analysis (equal); writing – review & editing (supporting). **Simon Anderson:** Formal analysis (supporting); writing – review & editing (supporting). **Vanessa Gwenin:** Writing – review & editing (supporting). **Christopher Gwenin:** Conceptualization (equal); formal analysis (equal); funding acquisition (equal); supervision (lead); writing – review & editing (supporting).

## ETHICS STATEMENT

None required.

## Figures and Tables

**FIGURE A1:**
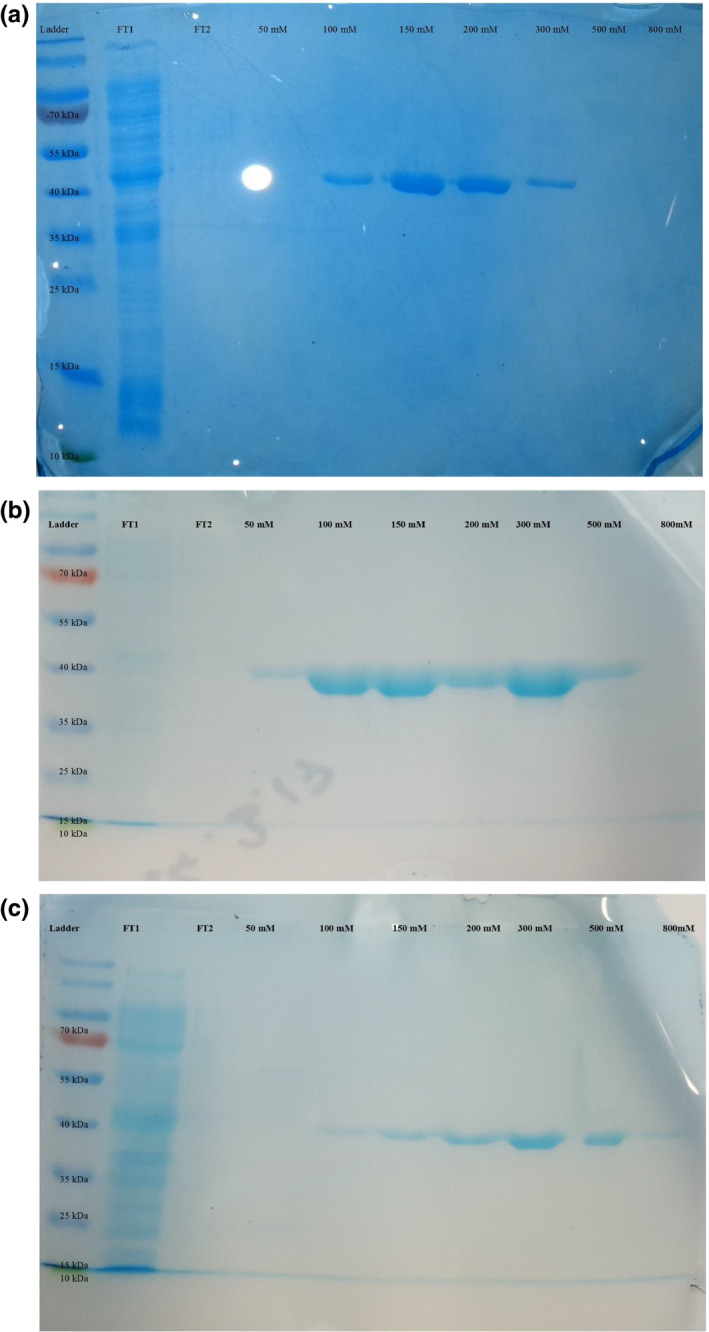
SDS‐PAGE of the purifications of (a) XenA‐his, (b) XenB‐his and (c) XenB‐cys. Flowthrough (FT) 1 and 2 were the first eluted fractions collected after adding the protein to the IMAC column. Imidazole concentration was then steadily increased from 50 mM to 800 mM to elute the pure his‐tagged proteins

## Data Availability

The data generated or analyzed during this study are included in this published article.
